# Multi-walled carbon nanotube-physicochemical properties predict the systemic acute phase response following pulmonary exposure in mice

**DOI:** 10.1371/journal.pone.0174167

**Published:** 2017-04-05

**Authors:** Sarah S. Poulsen, Kristina B. Knudsen, Petra Jackson, Ingrid E. K. Weydahl, Anne T. Saber, Håkan Wallin, Ulla Vogel

**Affiliations:** 1 National Research Centre for the Working Environment, Copenhagen Ø, Denmark; 2 Institute of Public Health, Copenhagen University, Copenhagen K, Denmark; 3 Department of Micro- and Nanotechnology, Technical University of Denmark, Kongens Lyngby, Denmark; Helmholtz Zentrum Munchen Deutsches Forschungszentrum fur Umwelt und Gesundheit, GERMANY

## Abstract

Pulmonary exposure to multi-walled carbon nanotubes (MWCNTs) has been linked to an increased risk of developing cardiovascular disease in addition to the well-documented physicochemical-dependent adverse lung effects. A proposed mechanism is through a strong and sustained pulmonary secretion of acute phase proteins to the blood. We identified physicochemical determinants of MWCNT-induced systemic acute phase response by analyzing effects of pulmonary exposure to 14 commercial, well-characterized MWCNTs in female C57BL/6J mice pulmonary exposed to 0, 6, 18 or 54 μg MWCNT/mouse. Plasma levels of acute phase response proteins serum amyloid A1/2 (SAA1/2) and SAA3 were determined on day 1, 28 or 92. Expression levels of hepatic *Saa1* and pulmonary *Saa3* mRNA levels were assessed to determine the origin of the acute phase response proteins. Pulmonary *Saa3* mRNA expression levels were greater and lasted longer than hepatic *Saa1* mRNA expression. Plasma SAA1/2 and SAA3 protein levels were related to time and physicochemical properties using adjusted, multiple regression analyses. SAA3 and SAA1/2 plasma protein levels were increased after exposure to almost all of the MWCNTs on day 1, whereas limited changes were observed on day 28 and 92. SAA1/2 and SAA3 protein levels did not correlate and only SAA3 protein levels correlated with neutrophil influx. The multiple regression analyses revealed a protective effect of MWCNT length on SAA1/2 protein level on day 1, such that a longer length resulted in lowered SAA1/2 plasma levels. Increased SAA3 protein levels were positively related to dose and content of Mn, Mg and Co on day 1, whereas oxidation and diameter of the MWCNTs were protective on day 28 and 92, respectively. The results of this study reveal very differently controlled pulmonary and hepatic acute phase responses after MWCNT exposure. As the responses were influenced by the physicochemical properties of the MWCNTs, this study provides the first step towards designing MWCNT that induce less SAA.

## Introduction

Multi-walled carbon nanotubes (MWCNTs) exhibit unique electrical, thermic and strengthening properties. But their increased production has also increased the potential risk of human exposure [1;2]. It is well established in rodent models that pulmonary exposure to MWCNTs through inhalation, instillation or aspiration is associated with lung inflammation, genotoxicity, fibrosis and granuloma formation [3–14]. In addition, pulmonary exposure to MWCNTs may increase the risk of developing cardiovascular diseases (CVD) [15]. Indeed, several rodent studies have shown that exposure to MWCNTs and single-walled carbon nanotubes (SWCNTs) induce CVD outcomes such as impaired vasodilation and increased plaque progression [16–19], just as it is well-established that pulmonary exposure to respirable air particulates is linked to increased risk of CVD [20–26]. Also, increased pulmonary expression and increased systemic levels of the acute phase response (APR) protein serum amyloid A (SAA) have been reported after pulmonary exposure to MWCNTs and other engineered nanomaterials (ENMs) [27–33]. Similar to the APR protein C-reactive protein (CRP), elevated plasma levels SAA is a risk factor for CVD in humans [34–37]. SAA (SAA1-4) is a highly conserved family of apolipoproteins associated with high density lipoproteins (HDL). However, species specific differences in the SAA isoforms and their expression exist. In humans, *Saa3* is only expressed in mammary gland epithelial cells [38], whereas *Saa1* and *Saa2* are expressed both hepatically and extra-hepatically [39]. In mice, *Saa3* is expressed in various tissues, including the lung, while *Saa1* and *Saa2* have previously been considered liver specific [40].

SAA proteins are secreted under cytokine control in response to local or systemic disturbances (e.g. infections) and can be induced over 1000-fold. During an acute phase response, SAA reaches circulation and replaces ApoA-1 as the major HDL protein, and thereby impairs HDL’s ability to mediate cholesterol efflux from macrophages [41–44]. As a result reverse cholesterol transport is reduced and peripheral cholesterol is sequestered. In addition, cholesterol is transported from HDL to macrophages. SAA-HDL thereby facilitates the transformation of macrophages into foam cells, which are a major component of fatty streaks observed during development of atherosclerosis [41;45]. Repeated pulmonary exposure by intratracheal instillation to recombinant human SAA, which is highly homologous to mouse SAA, induced both pulmonary inflammation and plague progression in female ApoE^−/−^ mice fed a Western-type diet (Daniel Christophersen et al, Unpublished results). This is consistent with the observation that overexpression of *Saa1* in ApoE^−/−^ mice was reported to increase plaque progression [46].

Hazard evaluation of MWCNTs as a group is problematic because of the tremendous variation in physicochemical properties, including diameter thickness, length, aspect-ratio, curvedness, purity, metal content and surface chemistry. Most studies investigating MWCNT-induced toxicity have assessed only one or few MWCNTs, and only few studies have attempted to correlate toxicity with physicochemical properties [8;9;31;47–49]. In a previous study, we assessed pulmonary inflammation and genotoxicity in the lungs of mice after exposure to a panel of 10 MWCNTs with the aim of associating toxicity endpoints with the physicochemical properties of the MWCNTs [8]. Using adjusted, multiple regression analyses, the specific surface area (BET) was identified as a positive predictor of pulmonary inflammation, such that a greater surface area resulted in more inflammation, whereas a larger diameter resulted in more DNA damage [8]. These results agree with previous published results, which emphasize the usability of the method and highlights its use as an alternative to the prevalent toxicity-testing approach.

We have previously found that pulmonary exposure to many different nanomaterials induces a pulmonary acute phase response [9;27;32;50]. In contrast, no hepatic acute phase response was identified after inhalation exposure to carbon black nanoparticles [51]. However, pulmonary exposure to a thin and short CNT was found to induce a strong hepatic acute phase response, in addition to the pulmonary acute phase response, whereas a thick and long MWCNT was less potent [31]. We therefore aimed at identifying physicochemical determinants of the hepatic and the pulmonary acute phase responses for MWCNT exposure. Plasma levels of the acute phase proteins CRP and SAA are predictors of CVD in epidemiological studies [37;52], and we therefore focused on identifying physicochemical predictors of acute phase response at the plasma level. The SAA isoforms were selected, as inflammatory stimuli only moderately induces CRP levels in mice [53;54]. Using a similar setup and same statistical methods as previously described [8], this study assesses systemic SAA3 and SAA1/2 protein levels after pulmonary exposure to a panel of 14 MWCNTs, for identifying physicochemical properties that were related to increased plasma SAA levels and consequently risk of developing CVD. Pulmonary *Saa3* and hepatic *Saa1* mRNA expression levels were measured to determine the origin of the systemic response. The 14 MWCNTs varied in length, diameter, surface area, functionalization level and metal content.

## Material and methods

This study consists of 3 parts with very similar, but not identical, experimental setups. An overview of the parts is presented in S1 Table. Part 2 was first performed, in which the doses 18, 54 and 162 μg MWCNT/mouse were used. We later decided on using lower doses for more relevant measurements, and therefore used doses 6, 18 and 54 μg MWCNT/mouse for part 1 and 3. There is therefore no 6 μg MWCNT/mouse dose for part 2. Similarly, post-exposure days for part 2 were 1, 3 and 28, whereas we later decided to use post-exposure days 1, 28 and 92 in part 1 and 3 to include a more chronic time point. To allow for comparison, the later time point (92 days) for NM-400 and NM-401 exposure was included in part 3. The experimental setups for part 1 and 2 have previously been published [8;9].

### Mice

Animal handling was previously described in detail [8;9]. Briefly, 5–7 week old female C57BL/6J BomTac (part 1 and 3) or C57BL/6J mice (part 2) were obtained from Taconic Europe (Ejby, Denmark). They were acclimatized for 1–3 weeks before the experiment. Average mice weight prior to experimentation was 19.2 g (SD = 1.24). All mice received food (1324 Altromin) and sterile water ad libitum during the whole experiment. The mice were group housed with 3–7 mice (part 1 and 3) or 10 mice (part 2) per cage in polypropylene cages with sawdust bedding and enrichment at controlled temperature 21 ± 1°C and humidity 50 ± 10% with a 12-h light/12-h dark cycle. Mice were identified by tail markings for the early euthanization time points (day 1 and 3) and by ear punch for the later (day 28 and 92).

### Materials

Fourteen MWCNTs were included in the study. Ten were purchased from Cheap Tubes (Brattleboro, VT, USA) and named NRCWE-040 to NRCWE-049. They were organized in three groups according to their physical properties as informed by the manufacturer (thin, thick, and short, group I-III, respectively), with each group (I-III) encompassing a pristine, a hydroxyl-, and a carboxy-functionalized type. In addition, group III included an amino-functionalized type. The physicochemical properties of these MWCNTs have previously been thoroughly characterized both in dry and in dispersed state [8;47]. The 4 remaining MWCNTs are from the OECD WPMN and were kindly donated by the European Union Joint Research Centre (Ispra, Italy), named NM-400 to NM-403. However, a different batch of NM-400 (called NRCWE-026) was used in part 2. With the exception of a greater content of aluminum in NRCWE-026 compared to NM-400, the two batches were very similar [47]. The name NM-400 will therefore be used for both NRCWE-026 and NM-400. Thorough characterization of NM-400 to NM-403 in dry form has previously been published [47]. A summary of the main physicochemical properties of the 14 MWCNTs are presented in Table 1. Crocidolite asbestos, a gift from Leibniz Research Institute for Environmental Medicine [55], and carbon black nanoparticles, Printex 90, a gift from Degussa-Hüls (today Evonic), Frankfurt, Germany, were included as reference materials.

**Table 1:** Overview of selected physicochemical characteristics of the studied MWCNT and reference materials.

**MWCNT group**	**Code**	**Type**	**Source**	**Product code**	**Length# nm (±SD)**	**Diameter# nm (±SD)**	**Oxygen content (mmol/g)**
**Group I**	**NRCWE-040**	PRISTINE	Cheaptubes	sku-030102	518.9 (±598)	22.1 (±7.8)	0.35
	**NRCWE-041**	OH	Cheaptubes	sku-030202	1005 (±2948)	26.9 (±10.1)	1.69
	**NRCWE-042**	COOH	Cheaptubes	sku-030302	723.2 (±971.9)	30.2 (±14.2)	4.09
**Group II**	**NRCWE-043**	PRISTINE	Cheaptubes	sku-030107	771.3 (±3471)	55.6 (±18.1)	0.18
	**NRCWE-044**	OH	Cheaptubes	sku-030207	1330 (±2454)	32.7 (±13.6)	0.23
	**NRCWE-045**	COOH	Cheaptubes	sku-030307	1553 (±2954)	30.2 (±15.6)	0.63
**Group III**	**NRCWE-046**	PRISTINE	Cheaptubes	sku-030111	717.2 (±1214)	29.1 (±16.1)	0.63
	**NRCWE-047**	OH	Cheaptubes	sku-030112	532.5 (±591.9)	22.6 (±10.1)	0.26
	**NRCWE-048**	COOH	Cheaptubes	sku-030113	1604 (±5609)	17.9 (±17.9)	0.58
	**NRCWE-049**	NH2	Cheaptubes	sku-030114	731.1 (±1473)	14.9 (±5.6)	0.33
**Standard materials**	**NM-400**‡	PRISTINE	OECD WPMNM	JRCNM04000a	847 (±446)	11 (±3)	0.79
	**NM-401**	PRISTINE	OECD WPMNM	JRCNM04001a	4048 (±2371)	67 (±24)	0.03
	**NM-402**	PRISTINE	OECD WPMNM	JRCNM04002a	1372 (±836)	11 (±3)	0.28
	**NM-403**	PRISTINE	OECD WPMNM	JRCNM04003a	443 (±222)	12 (±7)	0.19
**Reference materials**	**Printex 90**	-	Evonik	Printex 90	ND	9	ND
	**Crocidolite**	-	Leibniz IUF	-	90% < 4500£	90% < 460£	ND
**MWCNT group**	**Code**	**BET (m2/g)**	Fe* **content**	Co* **content**	Ni* **content**	Mg* **content**	Mn* **content**
**Group I**	**NRCWE-040**	150	0.2	0.001	0.56	0.01	0.002
	**NRCWE-041**	152	0.13	0.001	0.31	0.02	0.001
	**NRCWE-042**	141	0.08	0	0.21	0.03	0.001
**Group II**	**NRCWE-043**	82	0.008	0.001	1.2	0.01	-
	**NRCWE-044**	74	0.004	0.002	1.04	0.02	-
	**NRCWE-045**	119	1.17	0.25	1.34	0.02	0.002
**Group III**	**NRCWE-046**	223	0.008	0.25	0.0045	0.22	0.3
	**NRCWE-047**	216	0.007	0.25	0.0043	0.22	0.3
	**NRCWE-048**	185	0.007	0.24	0.0037	0.19	0.28
	**NRCWE-049**	199	0.004	0.25	0.0038	0.19	0.29
**Standard materials**	**NM-400**‡	254	0.2607	0.1063	0.0011	-	-
	**NM-401**	18	0.05	-	-	0.015	-
	**NM-402**	226	1.31	-	0.0011	0.001	0.001
	**NM-403**	135	0.002	1.2	0.0018	0.188	0.16
**Reference materials**	**Printex 90**	182	0.006	-	0.0003	-	-
	**Crocidolite**	5.24	7.23$	-$	-$	0.19$	0.03$

Detailed data published in Jackson et al. 2015.

* determined by WDXRF.

Chemical composition data were calculated wt% of the oxides of the elements determined.

All Fe is calculated as Fe^3+^.

^#^ determined by computerized image analysis of SEM micrographs and published previously (Poulsen et al 2016).

^‡^ NRCWE-26 contain 14.97% Al_2_O_3_ compared to 4.59% in NM-400.

^£^: Detailed data published by Muhle et al. 1987.

^$^: Meassured in 2% serum.

-: Not detected.

ND: Not determined.

### Material dispersion

The MWCNT- and Crocidolite samples were dispersed using the ENPRA dispersion protocol [56], either at 2.56 mg/ml stocks (part 1 and 3) or at 3.24 mg/ml stocks (part 2) in 0.45 μm MilliQ filtered Nanopure water with 2% (w/v) homologous mouse serum prepared in house. Printex 90 were dispersed at a 3.24 mg/ml stock in 0.45 μm MilliQ filtered Nanopure water as previously described [57]. Stocks were sonicated for 16 min in 4–6 ml volumes using a 400 W using a Branson Sonifier S-450D (Branson Ultrasonics Corp., Danbury, CT, USA) mounted with a disruptor horn, operated at 10% amplitude and cooled on ice water. Vehicle controls containing NanoPure water with 2% serum were sonicated as described for the MWCNT suspensions. Immediately before instillation, dilutions of stocks were sonicated again for 2 minutes.

### Exposure and tissue collection

Mice were instilled as previously described [58]. Briefly, mice were anesthetized at 8 weeks of age in a chamber using 4–5% isoflurane with a flow of 80% until fully relaxed. The mice were then dosed by a single intratracheal instillation of a 50 μl material suspension followed by 200 μl air with a 250 μl SGE glass syringe (250F-LT-GT, Micro- Lab, Aarhus, Denmark) according to the dose scheme in S1 Table. Number of mice was 6–7 per dose group and 12–33 mice per vehicle control group for each time point. The greater number of mice in control groups compared to dose groups is the result of the inclusion of 2–3 control animals per MWCNT material. This served two purposes: Due to the many MWCNT types involved, exposure had to be performed over several weeks. The inclusion of 2–3 control mice per MWCNT material enabled us to assess any possible day to day variance. In addition, with this setup we made sure to have enough control material for multiple experiments. Exposure group sizes were selected based on the experimentation method with the lowest power (Comet assay, previous publication [8]). After full recovery, the mice were transferred to the animal facility.

One, 3, 28 and 92 days after exposure, mice were euthanized by an I.P. injection with a ZRF cocktail (Zoletil Forte 250 mg, Rompun 20 mg/ml, Fentanyl 50 μg/ml in sterile isotone saline, dose 0.1 ml /25 gram bodyweight). Heart blood (800–1000 μl) was withdrawn via intracardiac puncture and stabilized with K_2_EDTA. It was then fractionated by centrifugation and plasma was collected and stored at -80°C. Bronchoalveolar lavage (BAL) fluid were collected as described previously [8]. Lung and liver tissue were collected, sectioned, snap-frozen in NUNC cryotubes in liquid nitrogen, and stored at -80°C. All procedures complied with the EC Directive 86/609/EEC and Danish law regulating experiments with animals (The Danish Ministry of Justice, Animal Experiments Inspectorate, permission 2006/561-1123).

### Total RNA extraction

RNA purification of lung tissue (16–22 mg) and liver tissue samples (13–17 mg) from mice 1 or 28 days after exposure to NM-400 to NM-403 was performed using the Maxwell 16 LEV simplyRNA tissue kit as specified by the manufacturer (Promega Biotech AB, Sweden). N = 3–6 per dose group. N = 3–15 for control groups. The RNA concentration after the extraction was measured on a NanoDrop 2000c Spectrophotometer (Thermo Fisher Scientific, Life technologies, Denmark). RNA samples showing A260/280 ratios between 1.9 and 2.15 were used in the experiment. Total RNA was stored at −80°C.

### cDNA synthesis

cDNA synthesis was performed using the TaqMan^®^ Reverse Transcription Reagents kit (ThermoFisher Scientific, Denmark), with total RNA as a template, as described in the manufacturer's protocol. A final RNA concentration of 10 ng/μl was used for each synthesis. The reactions were run on a PTC-100 Programmable Thermal Controller (MJ Research Inc., Canada), with a heating cycle of 25°C (10 min)/48°C (30 min)/95°C (5 min). The cDNA samples were stored at -20°C.

### Real-time qRT-PCR

The expression of hepatic serum amyloid A 1 (*Saa1*) and pulmonary serum amyloid A 3 (*Saa3*) were measured using a modified TaqMan Fast 2x Universal PCR Master Mix protocol (ThermoFisher Scientific, Denmark). For the *Saa1* analyses, a primer/probe mix was used (Mm00656927_g1, ThermoFisher Scientific, Denmark). For the *Saa3* analyses, a forward primer (140909J1C12: 5’-GCC TGG GCT GCT AAA GTC AT-3’), a reverse primer (40909J1B05: 5’-TGC TCC ATG TCC CGT GAA C-3’) and a *Saa3* probe (6-FAM- TCT GAA CAG CCT CTC TGG CAT CGCT –TAMRA) were used (all from TAG Copenhagen AS, Denmark). The samples were run in triplicates on 384-well reaction plates (Thermo Fisher Scientific, Denmark). Negative (minus reverse transcriptase), positive, and blank controls were included on each plate. The plates were run in the ViiA 7 Real-time PCR system (Thermo Fisher Scientific, Denmark). The relative expression was calculated using the Livak–Schmittgen method [59]. Samples deviating by two SD from the group mean were identified as outliers and were excluded from further analyses: From the lung analyses, we excluded 1 sample from the NM-401, day 1, dose 18 μg group, and 1 sample from the NM-401, day 28, dose 18 μg group. From the liver analyses, we excluded 1 sample from the NM-401, day 1, dose 54 μg group, and 1 sample from the NM-402, day 1, dose 54 μg group. The relative expression was normalized to the vehicle control levels for that MWCNT exposure. No significant difference was observed between the vehicle control groups and they were pooled within time points.

### Serum amyloid A1 and 2 protein levels in plasma

Plasma levels of serum amyloid A1 and 2 (SAA1/2) protein were measured after exposure to all MWCNTs at dose 54 μg and vehicle using the Tridelta PHASE^™^ Murine Serum Amyloid A ELISA Assay (BioRépair, Sinsheim, Germany) according to the manufacturer’s instructions. Only the highest dose was analyzed due to the cost of the kits and the large amounts of samples. In addition, when analyzing SAA3 plasma levels after exposure to the low and middle dose, we detected no increase compared to control, and we suspected similar results for SAA1/2 plasma levels. Three samples for each dose group were used (the 1, 3 and 5^th^ mouse from each group of 6–7 mice were chosen, whenever possible). Vehicle control groups contained 13 (day 1), 12 (day 28) and 12 (day 92) mice. SAA1/2 levels were normalized to vehicle control levels and are presented as a fold change.

### Serum amyloid A3 protein levels in plasma

Plasma levels of serum amyloid A3 (SAA3) protein were measured using sandwich ELISA from EDM Millipore (Cat. # EZMSAA#-12K) according to the manufacturer’s protocol. Three mice per dose group were selected (the 1, 3 and 5^th^ mouse from each group of 6–7 mice, whenever possible). Vehicle control groups contained 22 (day 1), 15 (day 28) and 10 (day 92) mice. SAA3 levels were normalized to vehicle control levels and are presented as a fold-change.

### Protein affinity of the plasma kit

Possible cross reactivity with the two SAA ELISA kits (Tridelta PHASETM Murine Serum Amyloid A ELISA Assay and sandwich ELISA from EDM Milipore) was analyzed using the standard material provided in the kits. A standard curve of each standard material was applied to the plates, which were processed and analyzed according to the manufacturer’s instructions.

### Statistical analyses

The statistical analyses were performed in SAS version 9.3 (SAS Institute Inc., Cary, NC, USA).

#### MWCNT-induced effects on endpoints

Variation attributed to differences in the 3 parts of the study was investigated using the PROC mixed protocol with part as a random effect. No effects of the different parts were observed. The effects of exposure and dose on pulmonary *Saa3* and hepatic *Saa1* mRNA expression were calculated using parametric two-way ANOVA, with a post hoc Tukey-type experimental comparison test for each separate time point. Not normally distributed data or data with inhomogeneous variance were log-transformed to reach parametric demands. The effects of exposure and dose on protein levels of SAA3 and SAA1/2 in plasma were analyzed using a parametric two-way (SAA3, day 1) and a parametric one-way (SAA3, day 28 and 92, and SAA1/2, all days) ANOVA, with a post-hoc Tukey-type experimental comparison test.

#### Analyses of pairwise associations

Pairwise correlations between the outcomes: Plasma protein levels of SAA3, plasma protein levels of SAA1/2 and pulmonary BAL neutrophil content were performed in Microsoft Excel (Microsoft. Redmond, Washington, Computer Software) and statistical significance was evaluated using Pearson Correlation analyses. A Pearson Correlation analysis was used to investigate the pairwise associations between physicochemical parameters (BET surface area, Fe, Mn, Ni, Co, Mg, diameter, length, and functionalization). We observed two clusters of highly correlated parameters (S2 Table). Cluster 1: BET surface area, diameter and Ni content. Cluster 2: Fe, Mn and Mg content. The parameters in these clusters could not be separated in the present dataset. Co content correlated with variables in with both cluster 1 and 2, however we chose to include it in cluster 2 due to its correlation with more variables in this cluster compared to cluster 1. In the multiple regression analyses we chose to use diameter (log-transformed) as the proxy variable for cluster 1 and Fe (log-transformed) as the proxy variable for cluster 2, as these explained most of the variation compared to the other variables in the clusters. In the Supplementary files, we also present the results using log-transformed BET, Ni and Mn content, since it was equally biologically relevant for the endpoints. OH-functionalization was chosen to represent oxygen content as a regression variable in the further analyses, as OH- and COOH-functionalization was determined by the same combustion elemental analysis [47]. Length and oxygen content did not correlate with the other parameters, and thus were the only independent variables.

#### Multiple regression analysis

Multiple regression analyses investigating the relationship between the remaining physicochemical properties (diameter, Fe, OH and length) and plasma protein levels of SAA3 and SAA1/2 were performed. Only time points showing significant difference between MWCNT and vehicle exposure were investigated in the multiple regression analyses. With the exception of BET surface area, all covariates were log-transformed using base 2, so that the estimated regression parameters showed the estimated effect corresponding to a doubling of the covariate. We used log(BET)/log(1.25) for BET surface area, so the estimated effect corresponded to a 25% increase in BET. As described previously, the detection limit was imputed for values below the detection limit of the chemical composition [8]. We included indicator variables for levels below detection limit, so the imputed values did not affect the estimated association between the outcome and the chemical composition. Statistical significance was determined at the 0.01 level in the multiple regression analyses, since no other correction for mass-significance was performed.

## Results

### Exposure characterization

Selected physicochemical characteristics for the 14 used MWCNTs, Crocidolite and Printex 90 are presented in Table 1. Surface modifications, specific surface area (BET), endotoxin, Fe_2_O_3_, CoO, NiO, MgO and MnO content (abbreviated to Fe, Co, Ni, Mg and Mn content) have been reported previously [47]. Average lengths varied from 443 to 4048 nm (Table 1), thus overall deviating from the lengths informed by the manufacturer. Length distributions for NRCWE-040 to NRCWE-049 have previously been published [8]. Data on length and diameter for NM-400 to NM-403 were obtained from the NANOGENOTOX joined action funded by EU Health Programme (n2009 21) [60]. NM-401 was both the longest and the thickest of the studied MWCNTs. As noted in earlier publications [8;47], oxygen content of the hydroxylated and carboxylated MWCNTs from group III was less than that of the pristine, which indicates no or very little functionalization.

### Hepatic *Saa1* expression

**Fig 1:**
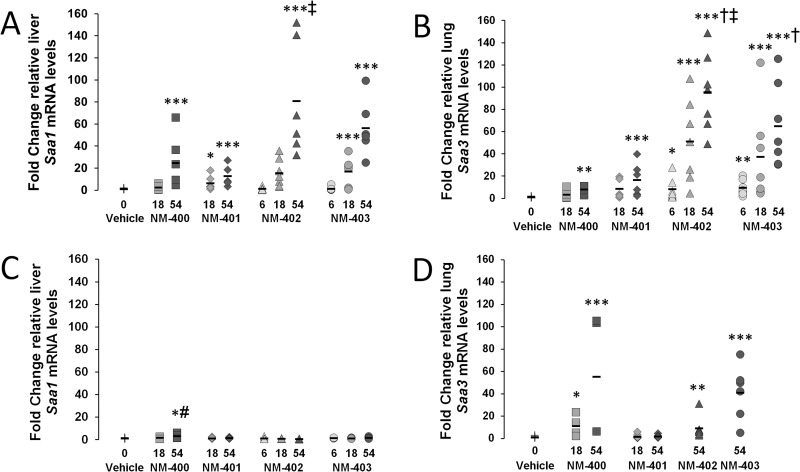
Changes in relative mRNA expression 1 or 28 days after exposure to the OECD standard material MWCNTs. Hepatic *Saa1* and pulmonary *Saa3* mRNA levels were normalized to *18S* and then normalized to vehicle control levels. A) Hepatic *Saa1* mRNA expression on day 1. B) Pulmonary *Saa3* mRNA expression on day 1. C) Hepatic *Saa1* mRNA expression on day 28. D) Pulmonary *Saa3* mRNA expression on day 28. *: p<0.05, **: p<0.01, ***: p<0.001 compared to vehicle controls. ‡: Significantly greater than NM-401 at the 54 μg/mouse dose. #: Significantly greater than NM-402 at the 54 μg/mouse dose. †: Significantly greater than NM-400 at the 54 μg/mouse dose.

**Fig 2:**
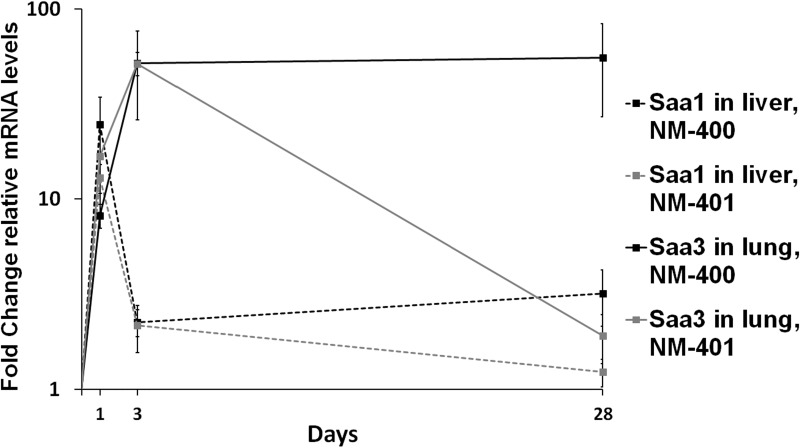
Time course for hepatic *Saa1* and pulmonary *Saa3* mRNA levels. Time points were 1, 3 and 28 days after exposure to standard materials NM-400 and NM-401. Error bars indicate SD.

The hepatic mRNA expression levels of *Saa1* were determined for NM-400 to NM-403 exposure on day 1 and 28 (Fig 1A and 1C) and for NM-400 and NM-401 on day 3 (S1 Fig and Fig 1). The hepatic *Saa1* expression was highest on day 1 and dose-dependency was observed for all assessed MWCNTs at this time point (Fig 1A). All MWCNTs significantly increased the hepatic *Saa1* expression after exposure to 54 μg/mouse, and NM-401 to NM-403 exposure additionally at 18 μg/mouse (Fig 1A). NM-402 induced a significantly greater response than NM-401 at 54 μg/mouse. *Saa1* expression was already lower and close to that of the vehicle controls on post exposure day 3 (S1 Fig), with only the 54 μg/mouse dose exposure to NM-400 and the 18 μg/mouse dose exposure to NM-401 resulting in increased expression compared to vehicle controls. On day 28, all expression levels had returned to control levels, except for the 54 μg/mouse dose exposure of NM-400, which was significantly increased compared to both controls and to NM-402 exposure (Fig 1C). Time course for hepatic *Saa1* expression across on days 1, 3 and 28 post-exposure to 54 μg/mouse NM-400 or NM-401 showed a similar pattern; highly increased expression on day 1, and with levels being at or close to that of the controls on day 3 and 28 (Fig 2).

### Pulmonary *Saa3* expression

The mRNA expression levels of *Saa3* in the lungs were determined for NM-400 to NM-403 exposure on day 1 and 28 (Fig 1B and 1D) and for NM-400 and NM-401 (S2 Fig) on day 3. Dose-dependency was observed for all MWCNTs on day 1 and all MWCNTs induced increased expression of *Saa3* after exposure to the 54 μg/mouse dose (Fig 1B). At this dose, NM-402 and NM-403 induced a significantly higher transcription level than NM-400, and NM-402 induced a significantly higher transcription level than NM-401. In addition, NM-402 and NM-403 exposure also induced significantly increased *Saa3* expression at the lower doses (6 and 18 μg/mouse). *Saa3* expression levels were higher on day 3 compared to day 1 (S2 Fig) and a similar dose dependency was observed. All exposures analysed significantly increased *Saa3* levels compared to the vehicle control, with the exception of the 18 μg/mouse dose exposure to NM-400, which was only borderline significant (P = 0.0629). On day 28, exposure to 54 μg/mouse of NM-400, NM-402 and NM-403, and 18 μg/mouse of NM-400, resulted in significantly increased *Saa3* expression, whereas levels for NM-401 exposure had returned to control levels (Fig 1D). Time course for pulmonary *Saa3* expression across days 1, 3 and 28 after exposure to 54 μg/mouse NM-400 or NM-401 are presented in Fig 2. Exposure to both types of MWCNTs resulted in similar time course on day 1 and 3, with the highest expression levels observed on day 3 (Fig 2). However, whereas exposure to NM-400 resulted to sustained increased expression 28 days after exposure, expression levels after NM-401 exposure had returned to that of the controls.

### Serum amyloid A 1 and 2 protein levels in plasma

**Fig 3:**
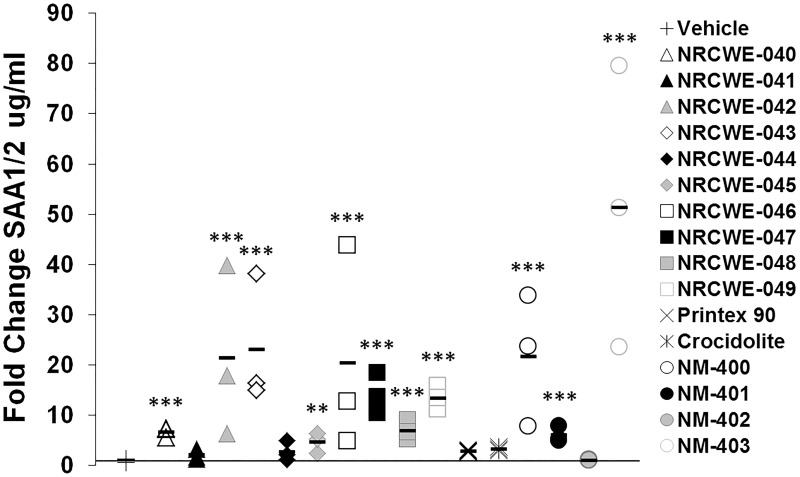
Fold change in SAA1/2 plasma protein levels 1 day after exposure to MWCNTs and reference materials. **: p<0.01, ***: p<0.001 compared to vehicle controls.

Although the SAA variants are very homologous, the antibody used to measure SAA1/2 levels was specific for SAA1/2, and antibody used for SAA3 was specific for SAA3, as no cross-reactivity was seen (S3 Table). We therefore distinguish between detection of SAA1/2 and SAA3. Plasma SAA1/2 levels were analyzed in vehicle control and the highest exposure groups on days 1, 28 and 92 (Fig 3 and S3 Fig). On day 1, all test materials induced significantly increased SAA1/2 levels, with the exception of NRCWE-041, NRCWE-044, Printex 90, Crocidolite, and NM-402 (Fig 3). No changes were observed on day 28 or 92 compared to vehicle controls (S3 Fig).

#### Analyses of pairwise associations

**Fig 4:**
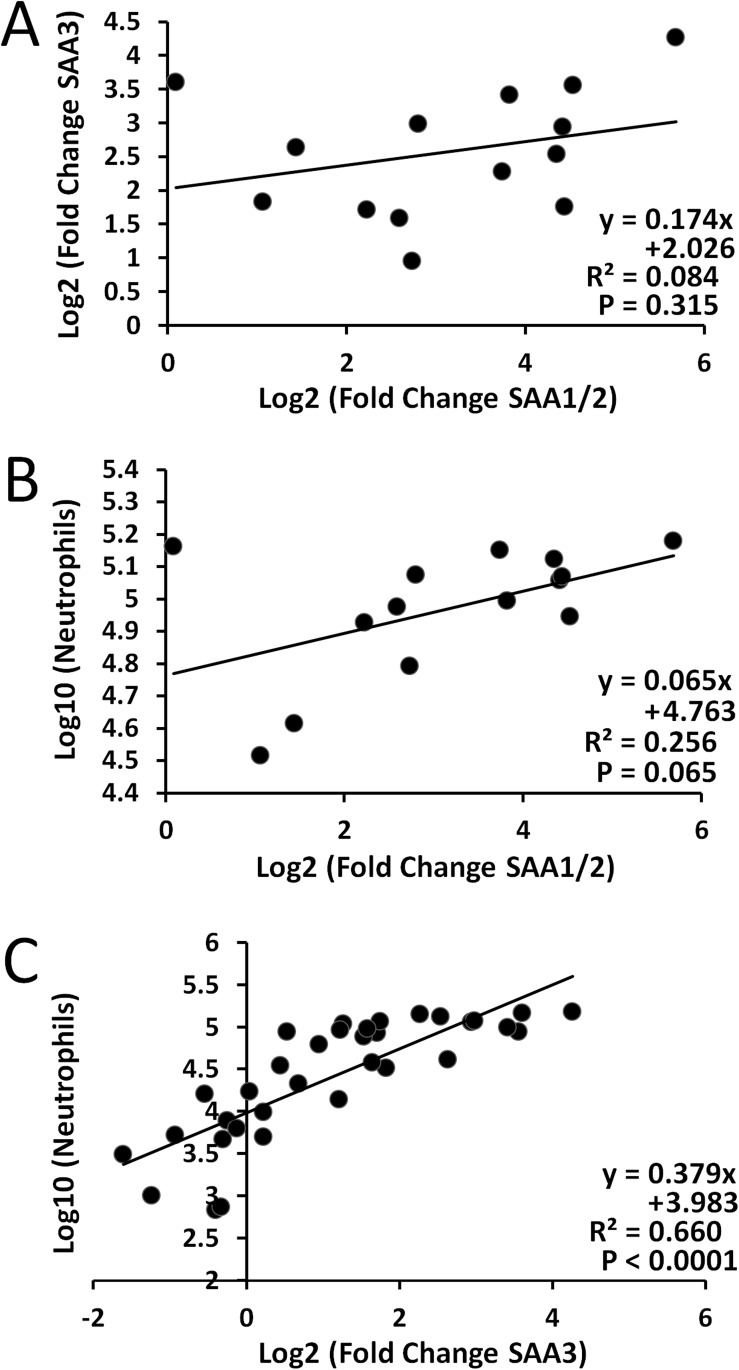
Correlations between plasma SAA1/2 protein levels, plasma SAA3 protein levels, and pulmonary inflammation on day 1. A) Transformed SAA1/2 protein vs. transformed SAA3 protein. B) Transformed SAA1/2 protein vs. transformed neutrophil influx. C) Transformed SAA3 protein vs. transformed neutrophil influx. Linear correlations are depicted in each graph.

Plasma SAA1/2 and SAA3 levels did not correlate (P = 0.315) (Fig 4A). Similar to this, plasma SAA1/2 levels only correlated at borderline with neutrophil influx in BAL fluid (P = 0.0651) (Fig 4B).

Before the multiple regression analyses, physicochemical parameters (BET surface area, diameter, length, Fe, Mn, Ni, Co, Mg, and oxygen content) were analyzed for pairwise associations in a Pearson correlation analysis. Length and oxygen content were the only independent variables (S2 Table). Two clusters of highly correlated parameters were observed: Cluster 1 (BET surface area, diameter and Ni content) and Cluster 2 (Fe, Mn, Co and Mg content). In the present dataset, the parameters in these clusters were inseparable. For the multiple regression analyses, diameter (log-transformed) and Fe content (log-transformed) were chosen as proxy variables for cluster 1 and 2, respectively, as these variables explained most of the variation in the analyses.

#### Multiple regression analysis

Plasma protein levels of SAA1/2 were not significantly different in exposed mice compared to vehicle controls on days 28 and 92, and these time points were therefore not analyzed for this endpoint. Of the remaining variables (diameter, length, Fe and OH content), increasing length significantly predicted decreasing plasma SAA1/2 protein levels (Table 2). No other variables were statistically significant. Multiple regression analyses using other proxy variables for cluster 1 and 2 are presented in S4 to S7 Tables.

**Table 2:** Multiple regression analyses.

**SAA1/2**					
**Day**	**Exposure Variable**	**Multiplicative Effect**	**LowerCL**	**UpperCL**	**Probt**
1	Per doubling in Diameter	1.052	0.676	1.637	0.817
	Per doubling in Fe_2_O_3_	0.929	0.833	1.035	0.176
	Per doubling in OH	0.894	0.712	1.123	0.327
	**Per doubling in Length**	**0.521**	**0.313**	**0.867**	**0.01**
**SAA3**					
**Day**	**Exposure Variable**	**Multiplicative Effect**	**LowerCL**	**UpperCL**	**Probt**
1	**Per doubling in Dose**	**1.053**	**1.046**	**1.059**	**<.0001**
	Per doubling in Diameter	0.899	0.733	1.103	0.303
	**Per doubling in Fe**_**2**_**O**_**3**_	**0.916**	**0.876**	**0.957**	**0.0001**
	Per doubling in OH	1.071	0.971	1.181	0.171
	Per doubling in Length	0.941	0.766	1.155	0.554
28	Per doubling in Diameter	0.741	0.566	0.97	0.03
	Per doubling in Fe_2_O_3_	0.983	0.92	1.05	0.608
	**Per doubling in OH**	**0.803**	**0.699**	**0.922**	**0.003**
	Per doubling in Length	1.348	0.989	1.838	0.059
92	**Per doubling in Diameter**	**0.66**	**0.518**	**0.84**	**0.001**
	Per doubling in Fe_2_O_3_	1.007	0.95	1.069	0.802
	Per doubling in OH	0.908	0.8	1.031	0.134
	Per doubling in Length	1.16	0.874	1.54	0.294

Physicochemical parameters and their influence on SAA1/2 and SAA3 protein content in the plasma after intratracheal exposure to MWCNT in a multiple regression analysis.

Significant p-values (P≤0.01) are highlighted in bold.

Multiple regression analysis was performed on day 1 only for SAA1/2 levels, as no significant changes from control levels were observed on day 28 and 92.

### Serum amyloid A 3 protein levels in plasma

**Fig 5:**
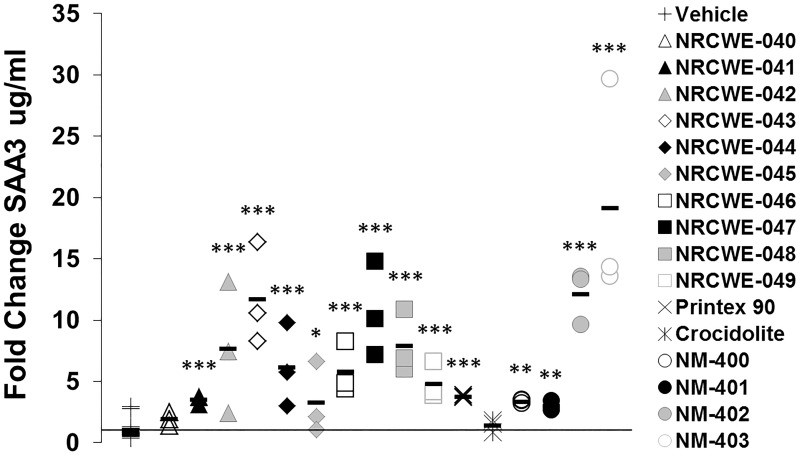
Fold change in SAA3 plasma protein levels 1 day after exposure to MWCNTs and reference materials. *: p<0.05, **: p<0.01, ***: p<0.001 compared to vehicle controls.

Plasma SAA3 levels were analyzed in vehicle control and 54 μg MWCNTs/mouse exposure groups (18 μg/mouse for Crocidolite and 162 μg/mouse for Printex 90) on days 1, 28 and 92 (Fig 5 and S4 Fig). With the exception of NRCWE-040, NRCWE-041 and Crocidolite, all test materials induced significantly increased SAA3 levels compared to controls 1 day after exposure (Fig 4). Therefore, lower doses (6 or 18 μg MWCNTs/mouse) were also included for NRCWE-040 to NRCWE-049. However, no increase in SAA3 levels were observed at these doses (data not shown). All SAA3 protein levels had returned to control levels 28 and 92 days after exposure, with the exception of protein levels after exposure to NM-401, which were significantly increased at both time points (S4 Fig). No trends across groups or functionalization types were observed.

#### Analyses of pairwise associations

As described earlier, there was no correlation was observed between plasma SAA3 and SAA1/2 levels (P = 0.315) (Fig 4A). Plasma SAA3 levels strongly correlated with neutrophil influx in the BAL fluid (P<0.0001), whereas SAA1/2 did not (Fig 4C). Diameter (log-transformed) and Fe content (log-transformed) were again chosen as proxy variables for cluster 1 and 2, respectively, in the multiple regression analyses.

#### Multiple regression analysis

Of the remaining variables (dose, diameter, length, Fe and OH content), increasing dose significantly predicted increased plasma SAA3 protein levels on day 1, whereas increasing Fe content (cluster 2) was protective (Table 2). On day 28, increasing oxygen content was protective of increased SAA3 plasma levels, while increasing diameter was protective on day 92. Multiple regression analyses using other proxy variables for cluster 1 and 2 are presented in S4 to S7 Tables.

## Discussion

The physicochemical properties of MWCNTs are important determinants of their toxic potential. Previous rodent studies have related MWCNT lengths, functionalization levels, and metal impurity content to MWCNT-induced adverse outcomes as inflammation, fibrosis and cancer [8;9;48;49]. Pulmonary exposure to MWCNTs and other ENMs has also been linked to increased risk of developing CVD [16;18;19;31]. We have proposed a mechanism for this increased risk by which pulmonary exposure to MWCNTs induces a strong pulmonary APR [61]. In mice, *Saa3* is the most upregulated APR gene in lung after ENM exposure, but *Saa1* and *Saa2* also have highly upregulated pulmonary expression [9;27;32;61]. Traditionally, circulating acute phase proteins are thought to be of hepatic origin, but we have reported that pulmonary exposure to Printex 90 carbon black nanoparticles induces a strong pulmonary APR, but little to no hepatic APR in mice [27;51]. In contrast, we have also shown that two different MWCNTs induced a hepatic APR in mice of varying potency following pulmonary exposure, even though the experimental protocol used was highly similar to that of the nano-carbon black study [31]. This indicates the involvement of different APR-related mechanisms after MWCNT and nano-carbon black exposure, which could be related to their physicochemical composition. However, very little data is available on the relationship between physicochemical properties of CNT and the APR, including SAA. We therefore assessed the systemic levels of SAA1/2 and SAA3 protein after pulmonary exposure to 14 MWCNTs in female C57BL/6J mice by intratracheal instillation to 3 doses (6, 18 or 54 μg/mouse for NRCWE-040 to NRCWE-049 and NM-402 to NM-403. 18 or 54 μg/mouse for NM-400 to NM-401) and 3 time points (1, 28 and 92 days post-exposure). Using a panel of 14 MWCNT, we were able perform comparisons not possible with the more standard setup of 1 or few MWCNTs seen in the literature.

Intratracheal instillation was chosen as dosing method as it offers precise, safe, cost-effective dosing with a fairly even distribution in the lung [8;62] and it is suitable for comparison of different materials. In addition, this method enables highly similar MWCNT dose levels, which is more difficult to obtain using inhalation. The doses correspond to a third, 1 or 3 times the expected 40-year exposure for workers at the recommended exposure limit of 1 mg carbon/m^3^ [63], when assuming 10% deposition [4], a ventilation rate of 1.8 l/h for mice, and a 40 h working week. Although the doses may appear relatively high, occupational CNT exposure concentrations of 30–300 mg/m^3^ have previously been reported [64–66]. The doses used were comparable to those of other MWCNT instillation/aspiration studies [7;30;67] and were selected to enable comparisons between both studies and across different ENMs [32;50;68–71].

Female mice were chosen to enable direct comparison with our previous nanomaterial studies. In addition, a cardio-protective effect of female sex hormones, particularly estrogen, has been proposed [72;73], which indicates that we could possibly have observed greater MWCNT-induced plasma SAA-level had we used male mice. In fact, a previous study investigating changes in SAA levels after MWCNT exposure using male C57BL/6J mice reported increased systemic SAA protein levels up to a year after exposure [30]. It should be note that the MWCNTs used in this study were longer (13.0±1.5 μm) than any of the MWCNTs used in the present study. Using female mice we avoided excessive changes related to the male sex. The APR was mainly assessed at the plasma protein level, as SAA is secreted into circulation and exerts its effects here, and as plasma levels of SAA is a predictor of CVD in epidemiological studies [37;52]. Expression of hepatic *Saa1* and pulmonary *Saa3* were assessed to address the origin of the circulating SAA proteins. The relationship between physicochemical properties of the MWCNTs and SAA protein in the plasma was analyzed by multiple regression analyses.

Printex 90 and Crocidolite asbestos were included as control material and to allow for comparison with previous studies [8;27;51;56;68]. Printex 90 exposure induced increased SAA3 levels, but not increased SAA1/2 levels (Figs 3 and 5). This is in concordance with previous studies, reporting a strong pulmonary APR, but little or no hepatic APR after Printex 90 exposure by inhalation and instillation [27;33;51]. Occupational exposure to asbestos has in large cohort studies been reported to increase the risk of developing CVD [74;75]. However, despite inducing pulmonary inflammation at all time points [8], no effects of Crocidolite exposure on SAA3 and SAA1/2 were observed (Figs 3 and 5). This is likely a consequence of the rather low dose of asbestos used (6 μg/mouse).

The 14 MWCNTs were acquired based on their physicochemical properties to ensure a diverse range of different MWCNT. All MWCNTs were thoroughly characterized in previous studies [8;9;47], and key physicochemical properties are presented in Table 1. Metal content, BET surface area, diameter thickness, and functionalization levels varied across both MWCNT groups and within groups (Table 1). Although the MWCNTs from group III were not properly functionalized (Table 1) [8], the remaining MWCNTs were, and the experimentally measured oxygen content of the MWCNTs were included as a parameter in the following multiple regression analyses. Average MWCNT lengths varied 9-fold from 443 to 4048 nm, with NM-401 being the longest. NM-401 shares many of its physical characteristics with the well-described Mitsui-7 [7;60], which has been classified as a possible carcinogen to humans (2B) by the IARC committee [76]. The remaining MWCNTs were relatively short (Table 1), there were few free tubes and most tubes were entangled and agglomerated [8;47]. The average MWCNT lengths in group I-III were similar with large standard deviations (Table 1). However, based on previously published size distributions of these MWCNTs [8], we noticed clear differences per MWCNT type in the number of single MWCNTs longer than 2 μm, such that MWCNTs with a larger average length had a greater number of long tubes. In addition, the 4 included OECD standard materials all exhibited more narrow standard deviations and more diversity in lengths (Table 1). We previously reported a batch-difference in the Al_2_O_3_ content of NM-400 [47], which could be important for their toxicity. However, the pulmonary *Saa3* mRNA levels and levels of SAA3 plasma protein after exposure to NM-400 were lower compared to exposure to NM-402 and NM-403 (Figs 1 and 5), which share many of the same physicochemical properties as NM-400, but a very low level of Al_2_O_3_. We therefore think that it is unlikely that the amount of Al_2_O_3_ present in NM-400 is enough to strongly bias the acute phase response after exposure.

The origin of proteins in plasma cannot easily be determined, since only mRNA levels can be used as evidence of synthesis. *Saa3*, *Saa1* and *Saa2* are all expressed in mouse lung tissue during a pulmonary acute phase response [9;27;29;70] with *Saa3* as the most differentially expressed *Saa* gene. In the mouse liver, *Saa1* and *Saa2* are the predominant *Saa* isoforms. We used *Saa3* mRNA levels in lung as a biomarker of the pulmonary acute phase response and *Saa1* mRNA levels as a biomarker for the hepatic acute phase response. All MWCNT types induced increased pulmonary *Saa3* expression on day 1 and 3, with the highest levels observed on day 3 (Fig 1B and S2 Fig). This time course of *Saa3* expression is very similar to those previously reported after pulmonary ENM exposure [9;27;32;61], which indicates a very general ENM-induced acute response with physicochemical-dependent variation in potency. Physical-related differences were observed on day 28; only the thin MWCNTs induced increased pulmonary *Saa3* expression (Fig 1D). In contrast to this, the hepatic expression of *Saa1*, representing the hepatic APR, was greatly induced on day 1, but diminished already on day 3 (Fig 2). Similar time course for hepatic *Saa1* has previously been described for Printex 90 exposure [27]. *Saa1* and *Saa2* expression were induced in the liver 3 days after exposure to two different MWCNTs [31] in a previous study, however, mainly at doses higher than used in the present study (162 μg/mouse). Only little or no increase was observed for the 18 and 54 μg/mouse doses [31]. This highlights an important difference between pulmonary and hepatic APR: Although both are induced quickly, the hepatic declines rapidly after its peak 1 day after exposure, whereas the pulmonary APR increases beyond this, peaks after several days and slowly abates.

SAA1/2 and SAA3 protein levels in plasma were measured using two different ELISA kits. No cross reactivity was detected with the standard murine SAA3 and SAA1/2 proteins and the ELISA assays were highly specific for their respective proteins (S3 Table). All MWCNT types induced increased plasma SAA3 levels on day 1, with the exception of NRCWE-040 and NRCWE-041 (Fig 5). In concordance with this, all the MWCNTs analyzed induced increased mRNA *Saa3* expression at this time point (Fig 1B). We have previously reported a significant correlation between pulmonary *Saa3* mRNA expression levels and plasma SAA3 proteins levels after MWCNT exposure [31], indicating that pulmonary-derived SAA3 proteins likely translocate to systemic circulation. In addition, we have reported greatly increased pulmonary *Saa3* expression, but low to no induction of hepatic *Saa3* expression after exposure to different ENMs [9;27;51]. A significant correlation between systemic SAA3 levels and pulmonary neutrophil influx was identified in this study (Fig 4C). We have previously reported a significant correlation between neutrophil influx in BAL and pulmonary *Saa3* mRNA levels after exposure to engineered nanomaterials (SWCNT, TiO_2_, CB and biofuel incineration plant dust) [32]. Similarly, the pulmonary cytokine expression profiles after exposure to two MWCNTs with very different physicochemical properties followed the profiles of pulmonary *Saa3* expression and of SAA3 levels in the blood [9;31], which supports the strong correlation between neutrophil influx and pulmonary *Saa3*/SAA3 levels observed in this study. This indicates that pulmonary exposure to MWCNTs leads to a significant size-dependent inflammatory response in the lungs and consequently increased pulmonary *Saa3* mRNA expression, leading to increased levels of SAA3 protein in blood.

SAA1 and SAA2 are considered liver-specific in humans [40], but we have previously documented increased *Saa1* and *Saa2* mRNA levels in mice lung following pulmonary exposure to ENMs [9;27]. However, given the size difference between lung and liver, the majority of plasma SAA1/2 is expected to be of hepatic origin. Almost every MWCNT type induced increased SAA1/2 proteins levels, but only on day 1. This concurs with hepatic *Saa1* expression, suggesting the liver as the primary source of plasma SAA1/2. Independent of the diameter and BET surface area, MWCNT length was significantly protective of SAA1/2 plasma protein increases in the multiple regression analysis on post-exposure day 1, such that a greater length would result in less SAA1/2 levels (Table 2). This suggests that designing longer MWCNT would help diminish their potential to induce SAA1/2 protein levels in the blood after exposure. Although large CNT lengths traditionally have been linked to increased MWCNT-induced toxicity [48;77;78], our previous studies have shown that a smaller (shorter and thinner) MWCNT induced more inflammation compared to a larger MWCNT [8;9]. Hepatic *Saa* expression is induced by IL6, IL1-β and TNF and thus, it may be induced following of systemic circulation of pulmonary expressed pro-inflammatory cytokines as previously suggested [33;79]. However, since SAA1/2 levels did not correlate with neutrophil influx or plasma content of SAA3 in the present study (Fig 4A and 4B), the induction of hepatic APR protein after MWCNT-exposure is likely regulated by other signaling pathways than the pulmonary acute phase response. Similar pulmonary inflammation-independent associations between ENM exposure and changes related to CVD have been reported earlier [24;80–82]. This contrast the proposed important role of pulmonary inflammation in the development of CVD by several studies [83–86]. However, instead of being mutually exclusive, these theories probably highlight different mechanisms in this highly multi-facetted disease.

Fe content was identified as protective of increased SAA3 proteins levels; such that a greater Fe content resulted in lower SAA3 levels (Table 2). However, as iron is a known ROS generator through the Fenton reaction [87] and oxidative stress plays a major part of the development of atherosclerosis [88;89], the observed protective effect of Fe is probably not due to the Fe content specifically. Instead it is driven by the tight covariance between Fe, Mg, Mn and Co (cluster 2) in this dataset (S2 Table), implicating that the effect is likely due to an effect of Mg, Mn or Co content, which all predicted increase SAA3 levels when analyzed without additional variables (data not shown). Although the present concentrations of Mg in the MWCNTs are unlikely to be toxic [90], both Mn and Co exposure has been related to adverse effects and could be possible predictors of increased systemic SAA3 protein levels [91–94]. Indeed, Co was linked to inflammation in our previous study [8], further emphasizing its role as a possible driver of systemic SAA3 induction. Although metal impurity content was determined, its localization in the MWCNTs and bioavailability is still unclear. Further studies investigating this aspect are needed to elucidate the true impact of metal impurities on MWCNT-induced toxicity.

Change in surface charge, -functionality and -reactivity, stability, and dispensability, can be obtained through MWCNTs surface functionalization, ultimately leading to altered toxicity [95–98]. We identified oxygen content (-OH and –COOH) as protective of increased systemic SAA3 protein levels on day 28 in the multiple regression analysis (Table 2). Interestingly, oxygen content was also identified as protective of pulmonary inflammation at the same time point in our previous study [8], again linking pulmonary inflammation and plasma SAA3 protein levels. Increased surface oxygen content may lead to more hydrophilic MWCNTs that are more evenly dispersed in the lung. In addition, structural defects introduced in the functionalization process may render the MWCNTs more susceptible to enzymatic and oxidative breakage. Such MWCNTs theoretically may be more easily degraded and thus more effectively cleared from the lungs, leading to attenuated pulmonary inflammation and hence also lowered SAA3 induction [96]. However, this needs to be confirmed experimentally.

The physical properties of the MWCNTs are likely important for their SAA-inducing potential. Diameter was identified as protective of increased SAA3 plasma levels on day 92, such that a larger diameter resulted in lowered SAA3 plasma levels (Table 2). This is similar to what was observed for inflammation at all time points [8], indicating a general lowered response of larger MWCNTs for these endpoints. However, only the OECD standard material NM-401, which is the only Mitsui MWCNT-7-like material in the panel, induced significantly increased SAA3 protein levels on day 28 and 92 (S4 Fig). These contrasting results are likely attributed to the large standard deviations observed for the standard materials NM-400 and NM-402 (S4 Fig).

As noted previously, no correlation was observed for inflammation and SAA1/2 protein levels (Fig 4B). Similarly, there was no correlation between SAA1/2 and SAA3 plasma levels (Fig 4A), indicating that these responses are not tightly related. Although it has been an underlying assumption that the APR is of hepatic origin [52;99], the impact of each type of response relative to each other has yet to be determined. This could possibly be achieved by identifying the type of SAA type primarily bound to HDL after MWCNT exposure, both at acute and at later time points. However, based on the observed large and sustained pulmonary *Saa3* increase, we consider the lung-derived APR to be an important part of the MWCNT-induced increased risk of developing CVD, especially at the later time points. More studies are still needed to fully elucidate the connection between pulmonary inflammation, the APR and CVD, and the mechanisms involved.

Using a panel of 14 MWCNTs, we attempted to identify specific physicochemical drivers of MWCNT-induced systemic SAA1/2 and SAA3 protein levels in a known mouse model. Based on the results of our analyses, designing MWCNTs with large diameters, and hence smaller surface area, low content of Mn and Co, and high levels of functionalization would reduce the risk of inducing SAA3-mediated increased risk of developing CVD after pulmonary exposure. These MWCNTs will induce less pulmonary inflammation and a lower pulmonary SAA3 production compared to other MWCNTs. This will lead to a larger proportion of HDL associated with ApoA-1 and less perturbation of the reverse cholesterol transport and less foam cell formation. In a similar fashion, designing MWCNTs with larger lengths would decrease the risk of inducing SAA1/2-mediated increased risk of developing CVD after pulmonary exposure. However, although a panel of 14 MWCNTs is large compared to the general number of MWCNT analyzed in the literature, the dataset is still small from a statistical point-of-view. The large covariance between parameters is a limitation of the study, and highlights the importance of careful interpretation of the results. Ideally, the hypotheses generated in this study should be tested using a larger array of MWCNTs. This would enable the determination of the SAA-inducing physicochemical properties of MWCNTs and thus the design of MWCNTs that are safer in regards to the SAA-induced risk of developing CVD. However, inflammation and risk of developing CVD are only one side of the many proposed adverse effects of MWCNT exposure. Pulmonary fibrosis and cancer has been reported to rely on a long length and fibrous shape of the MWCNTs [48;49;77;100]. It is therefore important to consider all aspects of MWCNT-induced toxicity, before labelling a MWCNT type as safe-by-design.

## Conclusion

Pulmonary exposure to MWCNTs induced dose-dependent pulmonary and hepatic acute phase responses. The pulmonary acute phase response was stronger in terms of fold increase and more long lasting than the hepatic acute phase response. Almost all of the 14 studied MWCNTs induced increased plasma levels of SAA3 and SAA1/2 protein on day 1. The OECD standard material NM-401 also induced significantly increased SAA3 levels on day 28 and 92. MWCNT length was identified as protective of increased SAA1/2 levels on day 1, such that a longer length results in lower SAA1/2 levels. Dose and content of Mn, Mg and Co predicted increased SAA3 protein levels on day 1, whereas oxidation and diameter of the MWCNTs were protective on day 28 and 92, respectively. Only SAA3 levels correlated with pulmonary neutrophil influx, and SAA1/2 and SAA3 protein levels did not correlate. The results of this study could provide the initial step towards designing MWCNTs that induce less SAA consequently less risk of inducing CVD following exposure.

## Supporting information

S1 Table Overview of the 3 parts included in the study. (DOCX)

S2 Table Pearson correlations of physicochemical parameters. All variables were log-transformed. Pairwise correlated parameters with correlation coefficiencies more than 0.75 or less than -0.75 are highlighted in bold. Red: Cluster 1. Blue: Cluster 2.
(DOCX)

S3 Table Cross analyses of ELISA kit affinity for SAA1/2 and SAA3 protein. —: Not determined.
(DOCX)

S4 Table Multiple regression analyses with diameter as proxy variable for cluster 1 and Mn as proxy variable for cluster 2. Physicochemical parameters and their influence on SAA1/2 and SAA3 protein content in the plasma after intratracheal exposure to MWCNT in a multiple regression analysis. Significant p-values (P≤0.01) are highlighted in bold. Multiple regression analysis was performed on day 1 only for SAA1/2 levels, as no significant changes from control levels were observed on day 28 and 92.
(DOCX)

S5 Table Multiple regression analyses with Ni as proxy variable for cluster 1 and Fe as proxy variable for cluster 2. Physicochemical parameters and their influence on SAA1/2 and SAA3 protein content in the plasma after intratracheal exposure to MWCNT in a multiple regression analysis. Significant p-values (P≤0.01) are highlighted in bold. Multiple regression analysis was performed on day 1 only for SAA1/2 levels, as no significant changes from control levels were observed on day 28 and 92.
(DOCX)

S6 Table Multiple regression analyses with BET as proxy variable for cluster 1 and Mn as proxy variable for cluster 2. Physicochemical parameters and their influence on SAA1/2 and SAA3 protein content in the plasma after intratracheal exposure to MWCNT in a multiple regression analysis. Significant p-values (P≤0.01) are highlighted in bold. Multiple regression analysis was performed on day 1 only for SAA1/2 levels, as no significant changes from control levels were observed on day 28 and 92.
(DOCX)

S7 Table Multiple regression analyses with BET as proxy variable for cluster 1 and Fe as proxy variable for cluster 2. Physicochemical parameters and their influence on SAA1/2 and SAA3 protein content in the plasma after intratracheal exposure to MWCNT in a multiple regression analysis. Significant p-values (P≤0.01) are highlighted in bold. Multiple regression analysis was performed on day 1 only for SAA1/2 levels, as no significant changes from control levels were observed on day 28 and 92.
(DOCX)

S1 Fig Fold change in relative hepatic *Saa1* expression after exposure to NM-400 and NM-401 on day 3. *Saa1* mRNA levels were normalized to *18S* and then normalized to vehicle control levels. **: p<0.01 compared to vehicle controls.
(DOCX)

S2 Fig Fold change in relative pulmonary *Saa3* expression after exposure to NM-400 and NM-401 on day 3. *Saa3* mRNA levels were normalized to *18S* and then normalized to vehicle control levels. **: p<0.01, ***: p<0.001 compared to vehicle controls.
(DOCX)

S3 Fig Fold change in plasma SAA1/2 levels after exposure to MWCNTs and reference materials on day 28 (A) and 92 (B). (DOCX)

S4 Fig Fold change in plasma SAA3 levels after exposure to MWCNTs and reference materials on day 28 (A) and 92 (B). ***: p<0.001 compared to vehicle controls.
(DOCX)

## Abbreviations

APR: Acute phase response
BAL: Bronchoalveolar lavage
BET surface area: Brunauer–Emmett–Teller specific surface area
CRP: C-reactive protein
CVD: Cardiovascular disease
ENMs: Engineered nanomaterials
HDL: High density lipoproteins
MWCNTs: Multi-walled carbon nanotubes
SAA: Serum amyloid A
SEM: Scanning Electron Microscopy
SWCNTs: Single-walled carbon nanotubes
