# Supplementary material for: Multi-walled carbon nanotube-physicochemical properties predict the systemic acute phase response following pulmonary exposure in mice
Source: PLoS One. 2017 Apr 5;12(4):e0174167. doi: 10.1371/journal.pone.0174167 (PMC5381870; doi:10.1371/journal.pone.0174167)
Supplement: S1 Table — (DOCX) [file pone.0174167.s001.docx]

**S1 Table. Overview of the 3 parts included in the study**

| **Part** | **Reference** | **Exposure** | **MWCNT** | **Day** | **Deposited dose (ug)** |
| --- | --- | --- | --- | --- | --- |
| **1** | Poulsen et al. 2016 | Intratracheal instillation | NRCWE-040 | 1, 28, 92 | 6, 18, 54 |
|  |  |  | NRCWE-041 | 1, 28, 92 | 6, 18, 54 |
|  |  |  | NRCWE-042 | 1, 28, 92 | 6, 18, 54 |
|  |  |  | NRCWE-043 | 1, 28, 92 | 6, 18, 54 |
|  |  |  | NRCWE-044 | 1, 28, 92 | 6, 18, 54 |
|  |  |  | NRCWE-045 | 1, 28, 92 | 6, 18, 54 |
|  |  |  | NRCWE-046 | 1, 28, 92 | 6, 18, 54 |
|  |  |  | NRCWE-047 | 1, 28, 92 | 6, 18, 54 |
|  |  |  | NRCWE-048 | 1, 28, 92 | 6, 18, 54 |
|  |  |  | NRCWE-049 | 1, 28, 92 | 6, 18, 54 |
|  |  |  | Crocidolite | 1, 28, 92 | 6, 18 |
|  |  |  | Printex 90 | 1, 28, 92 | 162 |
| **2** | Poulsen et al. 2015 | Intratracheal instillation | NRCWE-26 (NM-400) | 1, 3, 28 | 18, 54 |
|  |  |  | NM-401 | 1, 3, 28 | 18, 54 |
| **3** |  | Intratracheal instillation | NM-400 | 92 | 54 |
|  |  |  | NM-401 | 92 | 54 |
|  |  |  | NM-402 | 1, 28, 92 | 6, 18, 54 |
|  |  |  | NM-403 | 1, 28, 92 | 6, 18, 54 |
