# Supplementary material for: Multi-walled carbon nanotube-physicochemical properties predict the systemic acute phase response following pulmonary exposure in mice
Source: PLoS One. 2017 Apr 5;12(4):e0174167. doi: 10.1371/journal.pone.0174167 (PMC5381870; doi:10.1371/journal.pone.0174167)
Supplement: S2 Table — All variables were log-transformed. Pairwise correlated parameters with correlation coefficiencies more than 0.75 or less than -0.75 are highlighted in bold. Red: Cluster 1. Blue: Cluster 2. (DOCX) [file pone.0174167.s002.docx]

**S2 Table. Pearson Correlations of physicochemical parameters.**

|  | **Length** | **Diameter** | **BET** | **Ni** | **Co** | **Fe** | **Mn** | **Mg** | **OH** |
| --- | --- | --- | --- | --- | --- | --- | --- | --- | --- |
| **Length** | 1 | 0.4284 | -0.6507 | 0.1731 | -0.0980 | 0.3545 | -0.2982 | -0.3651 | -0.3793 |
| **Diameter** | 0.4284 | 1 | **-0.7654** | **0.8159** | -0.5998 | -0.0851 | -0.2385 | -0.1582 | -0.2808 |
| **BET** | -0.6507 | **-0.7654** | 1 | **-0.8106** | 0.5595 | 0.0098 | 0.4608 | 0.2702 | 0.6335 |
| **Ni** | 0.1731 | **0.8159** | **-0.8106** | 1 | **-0.7626** | 0.2083 | -0.6726 | -0.3941 | 0.1489 |
| **Co** | -0.0980 | -0.5998 | 0.5595 | **-0.7626** | 1 | -0.2717 | **0.7616** | **0.8516** | -0.0447 |
| **Fe** | 0.3545 | -0.0851 | 0.0098 | 0.2083 | -0.2717 | 1 | **-0.8885** | **-0.7585** | 0.2697 |
| **Mn** | -0.2982 | -0.2385 | 0.4608 | -0.6726 | **0.7616** | **-0.8885** | 1 | **0.8687** | -0.4952 |
| **Mg** | -0.3651 | -0.1582 | 0.2702 | -0.3941 | **0.8516** | **-0.7585** | **0.8687** | 1 | 0.1123 |
| **OH** | -0.3793 | -0.2808 | 0.6335 | 0.1489 | -0.0447 | 0.2697 | -0.4952 | 0.1123 | 1 |

**All variables were log-transformed. Pairwise correlated parameters with correlation coefficiencies more than 0.75 or less than -0.75 are highlighted in bold. Red: Cluster 1. Blue: Cluster 2.**
