# Supplementary material for: Multi-walled carbon nanotube-physicochemical properties predict the systemic acute phase response following pulmonary exposure in mice
Source: PLoS One. 2017 Apr 5;12(4):e0174167. doi: 10.1371/journal.pone.0174167 (PMC5381870; doi:10.1371/journal.pone.0174167)
Supplement: S3 Table — —: Not determined. (DOCX) [file pone.0174167.s003.docx]

**S3 Table. Cross analyses of ELISA kit affinity for SAA1/2 and SAA3 protein.**

| **Protein** | **Dose** | **Anti-SAA1/2** | **Anti-SAA3** |
| --- | --- | --- | --- |
| SAA1/2 | 1 | - | Not detected |
| SAA1/2 | 0.5 | 0.49 | Not detected |
| SAA1/2 | 0.25 | 0.25 | Not detected |
| SAA1/2 | 0.125 | 0.125 | Not detected |
| SAA1/2 | 0.0625 | 0.063 | Not detected |
| SAA3 | 2.2 | - | 2.068 |
| SAA3 | 1.1 | Not detected | 1.1 |
| SAA3 | 0.55 | 0.021 | 0.548 |
| SAA3 | 0.275 | Not detected | 0.276 |
| SAA3 | 0.1375 | 0.014 | 0.137 |
| SAA3 | 0.0688 | 0.008 | 0.077 |

**- : Not determined.**
