# Supplementary material for: Multi-walled carbon nanotube-physicochemical properties predict the systemic acute phase response following pulmonary exposure in mice
Source: PLoS One. 2017 Apr 5;12(4):e0174167. doi: 10.1371/journal.pone.0174167 (PMC5381870; doi:10.1371/journal.pone.0174167)
Supplement: S4 Table — Physicochemical parameters and their influence on SAA1/2 and SAA3 protein content in the plasma after intratracheal exposure to MWCNT in a multiple regression analysis. Significant p-values (P≤0.01) are highlighted in bold. Multiple regression analysis was performed on day 1 only for SAA1/2 levels, as no significant changes from control levels were observed on day 28 and 92. (DOCX) [file pone.0174167.s004.docx]

**S4 Table. Multiple regression analyses with diameter as proxy variable for cluster 1 and Mn as proxy variable for cluster 2.**

| **SAA1/2** | | | | | |
| --- | --- | --- | --- | --- | --- |
| **Day** | **Exposure Variable** | **Multiplicative Effect** | **LowerCL** | **UpperCL** | **Probt** |
| 1 | Per doubling in Diameter | 1.11 | 0.67 | 1.838 | 0.677 |
|  | Per doubling in MnO | 1.112 | 1.003 | 1.233 | 0.044 |
|  | Per doubling in OH | 0.964 | 0.763 | 1.218 | 0.753 |
|  | **Per doubling in Length** | **0.493** | **0.292** | **0.83** | **0.01** |
|  |  |  |  |  |  |
| **SAA3** | | | | | |
| **Day** | **Exposure Variable** | **Multiplicative Effect** | **LowerCL** | **UpperCL** | **Probt** |
| 1 | **Per doubling in Dose** | **1.051** | **1.044** | **1.058** | **<.0001** |
|  | Per doubling in Diameter | 0.944 | 0.706 | 1.263 | 0.695 |
|  | **Per doubling in MnO** | **1.066** | **1.018** | **1.115** | **0.007** |
|  | Per doubling in OH | 1.045 | 0.93 | 1.174 | 0.458 |
|  | Per doubling in Length | 0.901 | 0.719 | 1.13 | 0.363 |
|  |  |  |  |  |  |
| 28 | Per doubling in Diameter | 0.841 | 0.613 | 1.154 | 0.274 |
|  | Per doubling in MnO | 1.018 | 0.954 | 1.086 | 0.574 |
|  | **Per doubling in OH** | **0.79** | **0.682** | **0.914** | **0.003** |
|  | Per doubling in Length | 1.239 | 0.893 | 1.719 | 0.192 |
|  |  |  |  |  |  |
| 92 | **Per doubling in Diameter** | **0.695** | **0.527** | **0.917** | **0.012** |
|  | Per doubling in MnO | 0.989 | 0.936 | 1.045 | 0.695 |
|  | Per doubling in OH | 0.904 | 0.791 | 1.032 | 0.132 |
|  | Per doubling in Length | 1.079 | 0.807 | 1.443 | 0.598 |

**Physicochemical parameters and their influence on SAA1/2 and SAA3 protein content in the plasma after intratracheal exposure to MWCNT in a multiple regression analysis. Significant p-values (P≤0.01) are highlighted in bold. Multiple regression analysis was performed on day 1 only for SAA1/2 levels, as no significant changes from control levels were observed on day 28 and 92.**
