# Supplementary material for: Multi-walled carbon nanotube-physicochemical properties predict the systemic acute phase response following pulmonary exposure in mice
Source: PLoS One. 2017 Apr 5;12(4):e0174167. doi: 10.1371/journal.pone.0174167 (PMC5381870; doi:10.1371/journal.pone.0174167)
Supplement: S5 Table — Physicochemical parameters and their influence on SAA1/2 and SAA3 protein content in the plasma after intratracheal exposure to MWCNT in a multiple regression analysis. Significant p-values (P≤0.01) are highlighted in bold. Multiple regression analysis was performed on day 1 only for SAA1/2 levels, as no significant changes from control levels were observed on day 28 and 92. (DOCX) [file pone.0174167.s005.docx]

**S5 Table. Multiple regression analyses with Ni as proxy variable for cluster 1 and Fe as proxy variable for cluster 2.**

| **SAA1/2** | | | | | |
| --- | --- | --- | --- | --- | --- |
| **Day** | **Exposure Variable** | **Multiplicative Effect** | **LowerCL** | **UpperCL** | **Probt** |
| 1 | Per doubling in NiO | 0.956 | 0.891 | 1.025 | 0.195 |
|  | Per doubling in Fe_2_O_3_ | 0.934 | 0.85 | 1.027 | 0.155 |
|  | Per doubling in OH | 1.086 | 0.853 | 1.383 | 0.493 |
|  | **Per doubling in Length** | **0.353** | **0.21** | **0.594** | **0.0003** |
|  |  |  |  |  |  |
| **SAA3** | | | | | |
| **Day** | **Exposure Variable** | **Multiplicative Effect** | **LowerCL** | **UpperCL** | **Probt** |
| 1 | **Per doubling in Dose** | **1.052** | **1.046** | **1.058** | **<.0001** |
|  | Per doubling in NiO | 0.961 | 0.927 | 0.996 | 0.028 |
|  | **Per doubling in Fe_2_O_3_** | **0.937** | **0.894** | **0.983** | **0.009** |
|  | Per doubling in OH | 1.048 | 0.941 | 1.167 | 0.387 |
|  | Per doubling in Length | 0.95 | 0.759 | 1.189 | 0.652 |
|  |  |  |  |  |  |
| 28 | **Per doubling in NiO** | **0.917** | **0.884** | **0.952** | **<.0001** |
|  | Per doubling in Fe_2_O_3_ | 1.015 | 0.965 | 1.068 | 0.545 |
|  | Per doubling in OH | 0.914 | 0.803 | 1.04 | 0.166 |
|  | Per doubling in Length | 0.957 | 0.724 | 1.264 | 0.749 |
|  |  |  |  |  |  |
| 92 | **Per doubling in NiO** | **0.916** | **0.882** | **0.951** | **<.0001** |
|  | Per doubling in Fe_2_O_3_ | 1.051 | 0.998 | 1.107 | 0.058 |
|  | Per doubling in OH | 0.973 | 0.854 | 1.109 | 0.675 |
|  | Per doubling in Length | 0.892 | 0.671 | 1.184 | 0.418 |

**Physicochemical parameters and their influence on SAA1/2 and SAA3 protein content in the plasma after intratracheal exposure to MWCNT in a multiple regression analysis. Significant p-values (P≤0.01) are highlighted in bold. Multiple regression analysis was performed on day 1 only for SAA1/2 levels, as no significant changes from control levels were observed on day 28 and 92.**
