# Supplementary material for: Multi-walled carbon nanotube-physicochemical properties predict the systemic acute phase response following pulmonary exposure in mice
Source: PLoS One. 2017 Apr 5;12(4):e0174167. doi: 10.1371/journal.pone.0174167 (PMC5381870; doi:10.1371/journal.pone.0174167)
Supplement: S6 Table — Physicochemical parameters and their influence on SAA1/2 and SAA3 protein content in the plasma after intratracheal exposure to MWCNT in a multiple regression analysis. Significant p-values (P≤0.01) are highlighted in bold. Multiple regression analysis was performed on day 1 only for SAA1/2 levels, as no significant changes from control levels were observed on day 28 and 92. (DOCX) [file pone.0174167.s006.docx]

**S6 Table. Multiple regression analyses with BET as proxy variable for cluster 1 and Mn as proxy variable for cluster 2.**

| **SAA1/2** | | | | | |
| --- | --- | --- | --- | --- | --- |
| **Day** | **Exposure Variable** | **Multiplicative Effect** | **LowerCL** | **UpperCL** | **Probt** |
| 1 | Per 25% difference in BET | 0.826 | 0.698 | 0.979 | 0.029 |
|  | **Per doubling in MnO** | **1.16** | **1.046** | **1.287** | **0.006** |
|  | Per doubling in OH | 1.134 | 0.873 | 1.474 | 0.335 |
|  | **Per doubling in Length** | **0.412** | **0.253** | **0.673** | **0.001** |
|  |  |  |  |  |  |
| **SAA3** | | | | | |
| **Day** | **Exposure Variable** | **Multiplicative Effect** | **LowerCL** | **UpperCL** | **Probt** |
| 1 | **Per doubling in Dose** | **1.052** | **1.045** | **1.059** | **<.0001** |
|  | Per 25% difference in BET | 0.965 | 0.862 | 1.079 | 0.526 |
|  | **Per doubling in MnO** | **1.079** | **1.023** | **1.139** | **0.006** |
|  | Per doubling in OH | 1.068 | 0.933 | 1.223 | 0.336 |
|  | Per doubling in Length | 0.859 | 0.673 | 1.097 | 0.221 |
|  |  |  |  |  |  |
| 28 | Per 25% difference in BET | 1.012 | 0.901 | 1.136 | 0.842 |
|  | Per doubling in MnO | 1.02 | 0.95 | 1.095 | 0.577 |
|  | **Per doubling in OH** | **0.784** | **0.655** | **0.939** | **0.01** |
|  | Per doubling in Length | 1.172 | 0.838 | 1.639 | 0.343 |
|  |  |  |  |  |  |
| 92 | Per 25% difference in BET | 1.078 | 0.966 | 1.204 | 0.174 |
|  | Per doubling in MnO | 0.976 | 0.916 | 1.04 | 0.439 |
|  | Per doubling in OH | 0.846 | 0.716 | 0.999 | 0.049 |
|  | Per doubling in Length | 1.019 | 0.745 | 1.393 | 0.905 |

**Physicochemical parameters and their influence on SAA1/2 and SAA3 protein content in the plasma after intratracheal exposure to MWCNT in a multiple regression analysis. Significant p-values (P≤0.01) are highlighted in bold. Multiple regression analysis was performed on day 1 only for SAA1/2 levels, as no significant changes from control levels were observed on day 28 and 92.**
