# Supplementary material for: Multi-walled carbon nanotube-physicochemical properties predict the systemic acute phase response following pulmonary exposure in mice
Source: PLoS One. 2017 Apr 5;12(4):e0174167. doi: 10.1371/journal.pone.0174167 (PMC5381870; doi:10.1371/journal.pone.0174167)
Supplement: S7 Table — Physicochemical parameters and their influence on SAA1/2 and SAA3 protein content in the plasma after intratracheal exposure to MWCNT in a multiple regression analysis. Significant p-values (P≤0.01) are highlighted in bold. Multiple regression analysis was performed on day 1 only for SAA1/2 levels, as no significant changes from control levels were observed on day 28 and 92. (DOCX) [file pone.0174167.s007.docx]

**S7 Table. Multiple regression analyses with BET as proxy variable for cluster 1 and Fe as proxy variable for cluster 2.**

| **SAA1/2** | | | | | |
| --- | --- | --- | --- | --- | --- |
| **Day** | **Exposure Variable** | **Multiplicative Effect** | **LowerCL** | **UpperCL** | **Probt** |
| 1 | Per 25% difference in BET | 0.893 | 0.765 | 1.043 | 0.147 |
|  | Per doubling in Fe_2_O_3_ | 0.934 | 0.842 | 1.035 | 0.185 |
|  | Per doubling in OH | 0.975 | 0.758 | 1.253 | 0.837 |
|  | **Per doubling in Length** | **0.43** | **0.252** | **0.736** | **0.003** |
|  |  |  |  |  |  |
| **SAA3** | | | | | |
| **Day** | **Exposure Variable** | **Multiplicative Effect** | **LowerCL** | **UpperCL** | **Probt** |
| 1 | **Per doubling in Dose** | **1.053** | **1.047** | **1.059** | **<.0001** |
|  | Per 25% difference in BET | 1.037 | 0.964 | 1.115 | 0.328 |
|  | **Per doubling in Fe_2_O_3_** | **0.918** | **0.879** | **0.96** | **0.0002** |
|  | Per doubling in OH | 1.051 | 0.942 | 1.173 | 0.371 |
|  | Per doubling in Length | 0.966 | 0.771 | 1.211 | 0.765 |
|  |  |  |  |  |  |
| 28 | Per 25% difference in BET | 1.051 | 0.949 | 1.164 | 0.326 |
|  | Per doubling in Fe_2_O_3_ | 0.997 | 0.932 | 1.068 | 0.94 |
|  | **Per doubling in OH** | **0.774** | **0.656** | **0.913** | **0.003** |
|  | Per doubling in Length | 1.279 | 0.898 | 1.822 | 0.166 |
|  |  |  |  |  |  |
| 92 | Per 25% difference in BET | 1.077 | 0.976 | 1.189 | 0.134 |
|  | Per doubling in Fe_2_O_3_ | 1.03 | 0.966 | 1.098 | 0.363 |
|  | Per doubling in OH | 0.856 | 0.731 | 1.002 | 0.053 |
|  | Per doubling in Length | 1.095 | 0.781 | 1.535 | 0.591 |

**Physicochemical parameters and their influence on SAA1/2 and SAA3 protein content in the plasma after intratracheal exposure to MWCNT in a multiple regression analysis. Significant p-values (P≤0.01) are highlighted in bold. Multiple regression analysis was performed on day 1 only for SAA1/2 levels, as no significant changes from control levels were observed on day 28 and 92.**
