# Supplementary material for: Multi-walled carbon nanotube-physicochemical properties predict the systemic acute phase response following pulmonary exposure in mice
Source: PLoS One. 2017 Apr 5;12(4):e0174167. doi: 10.1371/journal.pone.0174167 (PMC5381870; doi:10.1371/journal.pone.0174167)
Supplement: S2 Fig — Saa3 mRNA levels were normalized to 18S and then normalized to vehicle control levels. **: p<0.01, ***: p<0.001 compared to vehicle controls. (DOCX) [file pone.0174167.s009.docx]

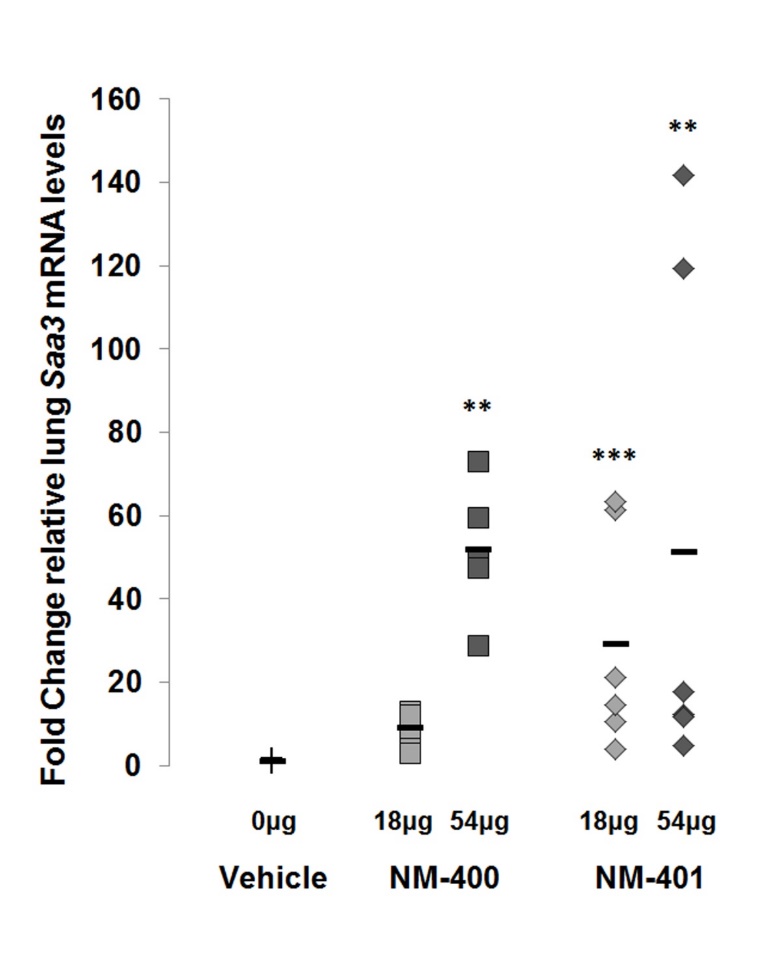


**S2 Fig. Fold change in relative pulmonary *Saa3* expression after exposure to NM-400 and NM-401 on day 3.** *Saa3* mRNA levels were normalized to *18S* and then normalized to vehicle control levels. **: p<0.01, ***: p<0.001 compared to vehicle controls.
