# Supplementary material for: Multi-walled carbon nanotube-physicochemical properties predict the systemic acute phase response following pulmonary exposure in mice
Source: PLoS One. 2017 Apr 5;12(4):e0174167. doi: 10.1371/journal.pone.0174167 (PMC5381870; doi:10.1371/journal.pone.0174167)
Supplement: S4 Fig — ***: p<0.001 compared to vehicle controls. (DOCX) [file pone.0174167.s011.docx]

**S4 Fig. Fold change in plasma SAA3 levels after exposure to MWCNTs and reference materials on day 28 (A) and 92 (B).** ***: p<0.001 compared to vehicle controls
